# Evaluation of the Clinical Efficacy of the Classic Prescription “Baihe Dihuang Decoction” Based on Meta-Analysis

**DOI:** 10.1155/2022/8559176

**Published:** 2022-09-12

**Authors:** Lei Peng, Xiao-Fei Zhang, Dong-Yan Guo, Bing-Tao Zhai, Yu-Jie Liang, Zhi-Ze Chen, Jun-Bo Zou, Ya-Jun Shi

**Affiliations:** Shaanxi Province Key Laboratory of New Drugs and Chinese Medicine Foundation Research, Pharmacy College, Shaanxi University of Chinese Medicine, Xianyang 712046, China

## Abstract

**Purpose:**

To explore the clinical application of Baihe Dihuang Decoction. To provide certain data support and theoretical basis for the clinical application of Baihe Dihuang Decoction in the future.

**Methods:**

With “Baihe Rehmannia Tang” as the search term, the search was carried out on CNKI, VIP, Wanfang, PubMed and other databases. The statistical analysis of Baihe Dihuang decoction for treating diseases was obtained. Meta-analysis of the data was performed using RevMan 5.3 software to analyze the main therapeutic indicators of the disease.

**Results:**

According to the 83 valid literature that can be found, it is shown that 17 are used for the treatment of depression, 14 are used for the treatment of menopausal syndrome, 24 are used for the treatment of insomnia, and 28 are used for the treatment of other diseases.

**Conclusion:**

In the treatment of depression, menopausal syndrome, and insomnia combined with Baihe Dihuang Decoction can have a better therapeutic effect and diminish the incidence of adverse reactions. It provides a theoretical basis for the study and experimental study of its active components.

## 1. Introduction

The development of classic traditional Chinese medicine prescriptions has gradually become one of the hot spots in the field of traditional Chinese medicine. The “classical prescriptions” of the “Supplementary Regulations Regarding the Administration of Printing and Distributing the Registration of Traditional Chinese Medicines” promulgated in 2008 refer to the prescriptions recorded in the medical records of the Qing Dynasty and before the Qing Dynasty that are still widely used, have clear curative effects, and have obvious advantages. Subsequently, the “Ancient Classic Famous Prescriptions Catalog (First Batch)” and the “Ancient Classic Famous Prescriptions Chinese Medicine Compound Preparation Simplified Registration and Approval Management Regulations” were successively released. The introduction of these policies has brought new opportunities to the research and development of the classical prescriptions. The first batch of classical prescriptions included Baihe Dihuang Decoction [1]. Baihe Dihuang Decoction originated from Synopsis of the Golden Chamber of the Golden Chamber by Zhang Zhongjing. Baihe Dihuang Decoction is composed of lily and rehmannia glutinosa, which has the effect of nourishing Yin, clearing heat and nourishing heart and lung.

Traditional Chinese medicine generally works in the body through multiple components-multiple targets-multiple pathways. Meta analysis can objectively evaluate the effect indicators, Heterogeneity and significance between different trial outcomes can also be explained, Therefore, we used evidence-based medicine to comprehensively search the published literature in both Chinese and English, collect the clinical trials of Baihe Dihuang Decoction, and discuss its therapeutic effects, so as to provide some evidence-based evidence for future clinical treatment. These provided theoretical basis for the one-step treatment of the disease.

## 2. Literature Analysis and Statistics

**Figure 1:**
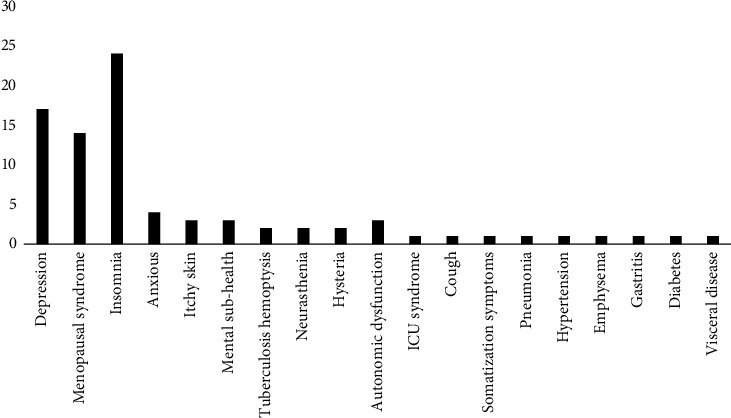
Number of literatures on related diseases.

With “Baihe Dihuang Decoction” as the key words, Wanfang, CNKI, VIP and PubMed databases were searched to screen out the literature of clinical use of Baihe Dihuang Decoction. A total of 155 literatures were retrieved and 83 literatures were obtained after deduplication, including 17 literature about depression, 14 literatures about menopausal syndrome, 24 literature about insomnia, 4 literatures about anxiety, 3 literatures about itchy skin, 3 literatures about mental sub-health, 2 literatures about *tuberculosis* hemoptysis, 2 literatures about neurasthenia, 2 literatures about hysteria, 3 literatures about autonomic dysfunction, 1 literature about icu syndrome, 1 literature about cough, 1 literature about somatization symptoms, 1 literature about pneumonia, 1 literature about hypertension, 1 literature about emphysema, 1 literature about gastritis, 1 literature about diabetes, 1 literature about visceral disease, as shown in Figure 1. It can be seen that Baihe Dihuang Decoction is mainly used clinically to treat depression, menopausal syndrome, and insomnia. Meta-analysis was used to evaluate the efficacy and safety of Baihedihuang Decoction in the treatment of depression, menopausal syndrome and insomnia, so as to provide evidence-based reference for clinical application.

## 3. Meta Analysis of the Main Clinical Indications of Baihe Dihuang Decoction

### 3.1. Meta Analysis and Methods

The key words “Baihe Dihuang Decoction” and “depression,” “Baihe Dihuang Decoction” and “menopause syndrome” or “climacteric syndrome,” “Baihe Dihuang Decoction” and “insomnia” were searched in Wanfang, CNKI, VIP and PubMed databases, and the retrieval time was from the database establishment to March 27, 2021. Two researchers independently searched, screened literatures, evaluated quality and extracted data according to the inclusion and exclusion criteria, and used RevMan 5.3 software for analysis. All relevant literature were downloaded to Endnote software for further discussion. Duplicate records were deleted, the full text was reviewed, and the title/abstract is considered to be thematic. The above work was carried out independently by two investigators. Conflicts were resolved through consensus and discussion.

### 3.2. Inclusion and Exclusion Criteria

We designed inclusion criteria: (1) The patients in the randomized controlled trial of Baihe Dihuang Decoction in the treatment of depression meet Chinese Classification of Mental Disorders (CCMD) vertion 2, 3, or Hamilton Depression Scale (HAMD), or WHO Quality of Life-100 (QOL-100), or obstetrics and gynecology (OG), or Kupperman (KMI), or psychiatry, or gynecology of Chinese medicine (GCM), or criteria for diagnosis and therapeutic effect (CDTE), or Guiding Principles for Clinical Research of New Chinese Medicines (GPCRNCM), or Hospital Anxiety and Depression Scaleor (HADS), Chinese Medicine Diagnostics (CMD), or Assessment of functional impairment (AFI). The patients in the randomized controlled trial of Baihe Dihuang Decoction in the treatment of menopausal syndrome meet Atrial Fibrillation Guide (AFG) vertion 2014, or Internal Medicine of Chinese Medicine (IMCM), or obstetrics and gynecology (OG), or guidelines for the diagnosis and treatment of common gynecological diseases in traditional Chinese medicine (GDTCGDTCM), or Guidelines for Prevention and Treatment of Type 2 Diabetes in China (GPTTDC) vertion 2013, or Psychiatry, or Guiding Principles for Clinical Research of New Chinese Medicines (GPCRNCM), or Criteria for diagnosis and therapeutic effect (CDTE), or gynecology of Chinese medicine (GCM), or The Diagnostic and Statistical Manual of Mental Disorders (DSM), or Practical Chinese medicine psychiatry (PCMP). Patients in a randomized controlled trial for the treatment of insomnia meet Hamilton Depression Scale (HAMD), or Pittsburgh sleep quality index (PSQI), or Asberg Side-effect Rating Scale for Antidepressant (ASRSA), or Chinese Classification of Mental Disorders (CCMD-3) vertion 3, or Guiding Principles for Clinical Research of New Chinese Medicines (GPCRNCM), or Research progress in the treatment of insomnia by TCM syndrome differentiation (RPTITSD), or criteria for diagnosis and therapeutic effect (CDTE), or Classification and judgment of constitution of traditional Chinese medicine (CJCTCM), or endocrinology, or urosurgery, or Internal Medicine of Chinese Medicine (IMCM), or Criteria for diagnosis and therapeutic effect of internal medicine (CDTEIM), or obstetrics and gynecology (OG). (2) All trials were randomized controlled trials. (3) The experimental group was treated with Baihe Dihuang Decoction or Baihe Dihuang Decoction combined with other drugs, and the control group was treated with conventional methods. (4) The outcome measure of each study must include at least one of the following indicators: The screening indicators for depression are HAMD, or the total effective rate (TER), or Incidence of adverse reactions (IAR), or KMI, or National Institutes of Health Stroke Scale (NIHSS), or Bathel Index, or QOL-100, or Symptoms of traditional Chinese medicine (STCM), or Social function evaluation (OHS), or Post-stroke depression (PSD), or Neurological deficit score (NDS), or PSQI, or Social dysfunction scale (SDSS), or 5-HT, or Norepinephrine (NE), or Follicle Stimulating Hormone (FSH), or Luteinizing hormone (LH), or Estradiol (E_2_). The screening indicators for menopausal syndrome are TER, or menopause-specific quality of life questionnaire (MENQOL), or Simpson-Angus scale (SAS), or Anxiety Self-Rating Scale (SDS), or PSQI, or Yin Deficiency and Fire Prosperity Syndrome (YDFPS), or KMI, or Quality of Life (QL), or Hamilton depression scale (HAMD), or Hamilton anxiety scale (HAMA), or Luteinizing hormone (LH), or follicle Stimulating Hormone (FSH), or Estradiol (E_2_), or Testosterone (T), or Prolactin (PRL), or Norepinephrine (NE), or 5-HT, or Nitric oxide (NO), or Endothelin-1 (ET-1), or calcitonin gene-related peptide (CGRP), or CD-3,4,8, or IL-2. The screening indicators for insomnia are TER, or Incidence of adverse reactions (IAR), or PSQI, or TCM symptom score (TSS), or sleeping time (ST), or Falling asleep time (FAT), or number of night wakes (NNW), or sleep depth (SD), or sleep efficiency (SE), or sleep quality (SQ), or Dreaminess or nightmares (DN), or Drowsiness, Lack of energy (LE), or HAMD, or HAMA, or Asberg Side-effect Rating Scale for Antidepressant (ASRSA), or glycosylated serum protein (GSP). The exclusion criteria were designed as follows: (1) References, such as reviews, case reports, animal experiments, reviews that were considered irrelevant to the subject. (2) Diagnostic standard in statement was ambiguous. (3) The intervention of patients was not based on Baihe Dihuang Decoction. (4) Provide incomplete information and duplicate literatures.

### 3.3. Data Selection and Quality Assessment

The literature that met the requirements was screened, and the information, including author, year of publication, sample size, intervention and measurement results, was tabulated. The quality of the included studies was independently evaluated by two investigators according to the Cochrane Intervention System Evaluation Manual. Disagreements were resolved by consensus. The quality assessment is as follows: random sequence generation (selection bias), allocation concealment (selection bias), blinding of participants and personnel (performance bias), blinding of outcome assessment (detection bias), incomplete outcome data (attrition bias), selective reporting (reporting bias) and other bias. Each semester is judged at three levels. The “low risk” of prejudice means that the description of the method or procedure is adequate. An inadequate or incorrect description of a method or procedure means “High risk,” while the absence of a description of a method or procedure means “unclear risk.”

### 3.4. Data Analysis

We analyzed the data using Review Manager 5.3 (Cochrane Collaboration). Outcome measures such as TER were treated as dichotomous variables and emerged as the odds ratio (OR) with 95% conﬁdence intervals (95% CI). We evaluated the heterogeneity between the studies by using *Q* statistics and *I*^2^ tests. The data with low heterogeneity (*P* ≥ 0.1% and *I*^2^ ≤ 50%) were analyzed by using a ﬁxed-eﬀects model, while the data with high heterogeneity (*P* < 0.1 or *I*^2^ > 50%) were estimated by using the random-eﬀects model. Funnel plots reveal potential publication bias.

### 3.5. Meta Screening Results

**Figure 2:**
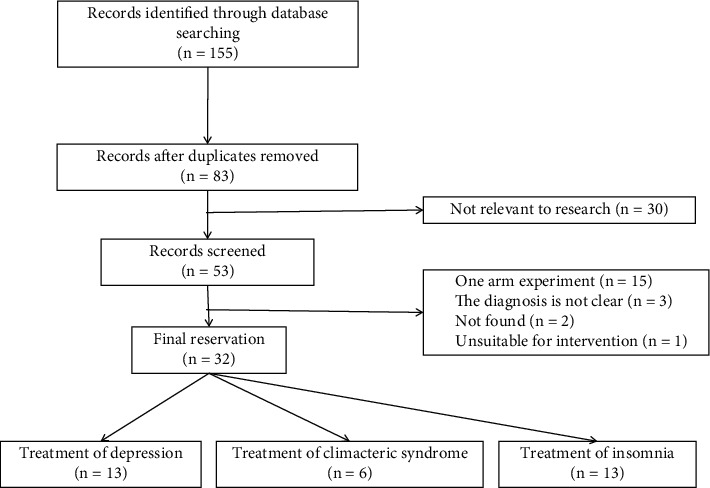
Literature screening flow chart.

After the database search, 155 articles were identified, of which 72 duplicate articles were deleted. 30 were excluded because of thematic disqualiﬁcation. In the process, 21 studies were excluded for the following reasons: 2 articles could not be found, 15 articles are single-arm designs, 3 studies with unclear diagnosis, 1 article is not suitable for intervention. 32 articles were eventually included. Among them, 13 [2–14] are on the treatment of depression. 6 [15–20] articles on treatment of menopausal syndrome. 13 [21–33] articles on the treatment of insomnia, as shown in Figure 2.

A total of 878 patients (451 cases in the experimental group and 427 cases in the control group) were enrolled in Baihedihuang decoction for depression treatment. The age of the patients ranged from 29 to 85years old, and there was no significant difference between the two groups by sex or sex. All trials were conducted before March 27, 2021, All reports are about comparing conventional treatment and conventional treatment combined with Baihe Dihuang Decoction treatment. In eligible trials, the conventional treatment plan is slightly different. Conventional antidepressants are generally psychotropic drugs and 5-HT, NE reuptake inhibitors. In some cases, Ganmai Dazao Decoction, Ginkgo biloba, etc are used. 13 studies reported treatment durations ranging from 2 weeks to 8 weeks. Baihe Dihuang Decoction for the treatment of menopausal syndrome selected 579 patients (290 cases in the test group, 289 cases in the control group), and the age of the patients was 40 to 60 years old. All reports are about comparing conventional treatment and conventional treatment combined with Baihe Dihuang Decoction treatment. In eligible trials, the conventional treatment plan is slightly different. Conventional treatment is generally given with hormonal drugs, and in some cases, hypoglycemic drugs, Huanglian Ejiao Decoction, etc are also given.The duration of treatment is 4 weeks to 12 weeks. Baihe Dihuang Decoction for the treatment of insomnia selected 1086 patients (554 cases in the test group, 532 cases in the control group). The age of the patient is 30 to 87 years old, and there was no significant difference between the two groups by sex or sex. All trials were conducted before March 27, 2021, All reports are about comparing conventional treatment and conventional treatment combined with Baihe Dihuang Decoction treatment. In eligible trials, the conventional treatment plan is slightly different. Conventional treatment drugs mostly use sedative, hypnotic and anxiolytic drugs, and in some cases Suanzaoren Decoction, acupuncture, etc are used. The duration of treatment is 2 to 8 weeks, as shown in Tables 1 and 2.

## 4. Depression

### 4.1. Quality of Included Trials

**Figure 3:**
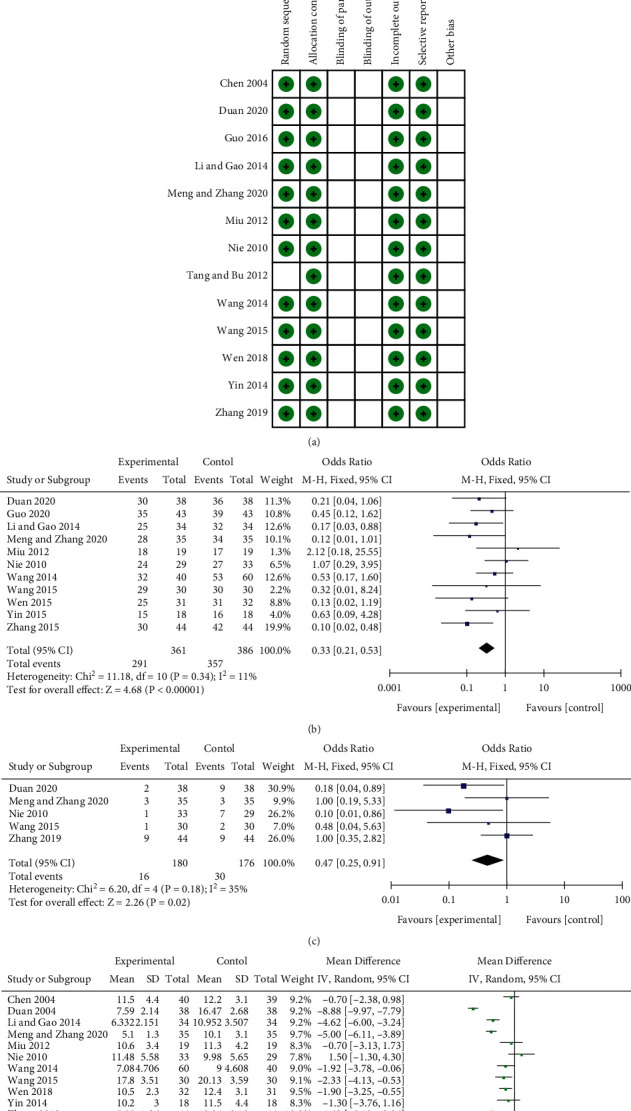
Depression correlation charts. Note: (a) Bias assessment risk of the study. Red circle, high bias risk; green circle, low bias risk; blank, unclear bias risk. (b) Forest chart of the total effective rate in the treatment of depression. (c) Forest chart of incidence of adverse reactions in treatment of depression. (d) Forest plot of HAMD value in treatment of depression.

According to Cochrane's risk of bias estimates, 12 trials mentioned randomly assigned participants and 1 did not mention it. None of the studies mentioned blindness of subjects and outcome evaluation. All of the literature had a low risk of allocation concealment, selective reporting and data integrity, as shown in Figure 3(a).

### 4.2. The Results of the Analysis Are Measured

#### 4.2.1. Total Effective Rate and Incidence of Adverse Reactions of Conventional Treatment Drugs and Conventional Treatment Drugs Combined with Baihe Dihuang Decoction in the Treatment of Depression

Judgement criteria for the total effective rate: The curative effect is determined according to the “Diagnosis and Curative Effect Criteria for Diseases and Syndromes of Traditional Chinese Medicine” issued by the State Administration of Traditional Chinese Medicine in 1994. Cure: The symptoms disappear, the spirit is normal: Effective: the symptoms are alleviated, and the mood is stable; Ineffective: there is no improvement in the mental and physical symptoms. Healed and effective are included in the total effective. The overall effective rate refers to the proportion of patients receiving rehabilitation and efficacy evaluation in the total group. 11 articles reported the total effective rate. Meta-analysis using a fixed-effect model (*P*=0.34, *I*^2^ = 11%) showed that combined Baihe Dihuang Decoction could significantly augment the efficacy of depression treatment (MD = 0.33, 95%CI: 0.21, 0.53; *P* < 0.00001), as shown in Figure 3(b). Five studies provided a description of the incidence of adverse reactions after conventional treatments combined with Baihe Dihuang Decoction, such as nausea, constipation, drowsiness, fatigue and dizziness. Meta-analysis using a fixed-effect model (*P* = 0.18, *I*^2^ = 35%) showed that combined Baihe Dihuang Decoction could reduce the incidence of adverse reactions in the treatment of depression. (MD = 0.47, 95%CI:0.25, 0.91; *P* = 0.02), as shown in Figure 3(c).

#### 4.2.2. HAMD of Conventional Treatment Drugs and Conventional Treatment Drugs Combined with Baihe Dihuang Decoction in the Treatment of Depression

HAMD is an important indicator reflecting the severity of depression in patients included in studies. 11 studies reported the detection of HAMD. Meta-analysis using random-effects model (*P*  <  0.00001, *I*^2^ = 93%) showed that combined Baihe Dihuang Decoction could significantly diminish HAMD in the treatment of depression (MD = −2.93, 95%CI: −4.56, −1.30; *P* = 0.0004), as shown in Figure 3(d).

#### 4.2.3. Other Score Indexes of Conventional Treatment Drugs and Conventional Treatment Drugs Combined with Baihe Dihuang Decoction in the Treatment of Depression

KMI, NIHSS, ADL, QOL-100, Symptoms of traditional Chinese medicine (Anxiety Somatization (AS), sleep disorder (SD), Despair), OHS, PSD, NDS, PSQI, SDS are various scoring indicators used to detect the degree of depression in patients. 2 studies mentioned the measurement of KMI. 2 studies mentioned the measurement of NIHSS. 2 studies mentioned the measurement of ADL. 1 study mentioned the measurement of QOL-100. 1 study mentioned the measurement of STCM (AS, SD, Despair). 1 study mentioned the measurement of OHS. 1 study mentioned the measurement of PSD. 1 study mentioned the measurement of NDS. 1 study mentioned the measurement of PSQI. 1 study mentioned the measurement of SDSS. Their MD and 95% confidence intervals are (MD = −5.41, 95%CI:−10.28, −0.54), (MD = −7.18, 95%CI: −8.88, −5.48), (MD = 3.83, 95%CI: −7.84, 15.50), (MD = 14.79, 95%CI:13.22, 16.36), (MD = −1.92, 95%CI: −2.45, −1.39), (MD = −2.17, 95%CI: −2.45, −1.88), (MD = −0.08, 95%CI: −0.32, 0.17), (MD = −0.81, 95%CI: −1.41, −0.21), (MD = 0.32, 95%CI: 0.09, 1.13), (MD = 0.20, 95%CI: −1.93, 2.33), (MD = −2.70, 95%CI: −3.15, −2.25), (MD = −3.50, 95%CI: −4.31, −2.69). Among them, NIHSS, QOL-100, AS, SD, OHS, PSQI and SDSS had significant changes, as shown in Table 3. It shows that compared with the conventional medication, the conventional medication combined with Baihe Dihuang Decoction has improved the symptoms of depression.

#### 4.2.4. Comparison of Neuroendocrine Function Improvement of Conventional Treatment Drugs and Conventional Treatment Drugs Combined with Baihe Dihuang Decoction in the Treatment of Depression

Analysis indicators are 5-HT, NE, FSH, LH and E_2_. 2 studies mentioned the determination of 5-HT, and analysis showed that the content of 5-HT was elevated (MD = 33.80, 95% CI:15.60, 51.99). 2 studies mentioned the determination of NE, and its content was significantly aumented (MD = 20.70, 95%CI: 12.66, 28.74, *P* < 0.00001). 1 study mentions the measurement of FSH, and its level is diminished (MD = −1.10, 95%CI: −16.91, 14.71). 1 study mentions the determination of LH (MD = 0.30, 95%CI: −7.36, 7.96). 1 study mentioned the measurement of E_2_, the level of E_2_ diminished (MD = 3.90, 95%CI: −8.52, 16.32), as shown in Table 4.

## 5. Menopausal Syndrome

### 5.1. Quality of Included Trials

**Figure 4:**
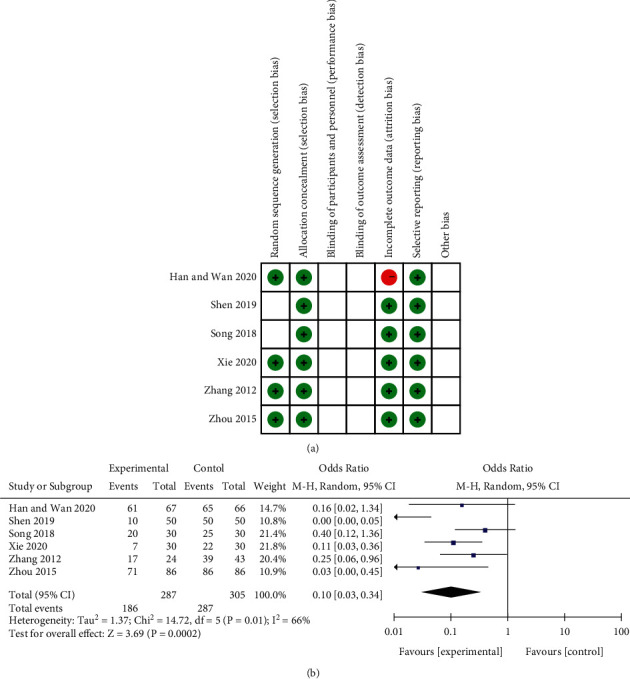
Menopausal syndrome correlation charts. Note: (a) Bias assessment risk of the study. Red circle, high bias risk; green circle, low bias risk; blank, unclear bias risk. (b) Other scoring indicators used to evaluate menopausal syndrome.

According to Cochrane's risk of bias estimates, 4 trials mentioned randomly assigned participants and 2 did not mention it. None of the studies mentioned blindness of subjects and outcome evaluation. 5 trials had low data integrity risks. 6 articles had a low risk of allocation concealment and selective reporting, as shown in Figure 4(a).

### 5.2. Analytical Result Measurement

#### 5.2.1. The Total Effective Rate of Conventional Treatment Drugs and Conventional Treatment Drugs Combined with Baihe Dihuang Decoction in the Treatment of Menopausal Syndrome

Judgement criteria for overall efficiency: Hot flashes, sweating, irritability, insomnia, palpitation and other symptoms disappear, and the color and volume during menstruation are normal, which means recovery; It is effective if symptoms such as hot flashes, sweating, irritability, insomnia and palpitation are diminished by more than half; If symptoms such as hot flashes, sweating, irritability, insomnia and palpitation are not enhanced or worsened, it is invalid. Healed and effective are included in the total effective. The total effective rate refers to the proportion of patients receiving rehabilitation and efficacy evaluation in the total group. 6 articles reported the total effective rate. Random effects model (*P* = 0.01, *I*^2^ = 66%) meta-analysis results showed that combining Baihe Dihuang Decoction in the treatment of menopausal syndrome could significantly enhance the efficacy (MD = 0.10, 95%CI: 0.03, 0.34; *P* = 0.0002), as shown in Figure 4(b).

#### 5.2.2. Other Score Indexes of Conventional Treatment Drugs and Conventional Treatment Drugs Combined with Baihe Dihuang Decoction in the Treatment of Menopausal Syndrome

MENQOL (BC, psychological, Body, Sex), SAS, SDS, PSQI, Yin Deficiency and Fire Prosperity Syndrome (YDFPS), KMI, QL, HAMD, HAMA are various scoring indicators used to detect the degree of menopausal syndrome in patients. 1 study mentioned the measurement of MENQOL (BC, psychological, Body, Sex) (MD = −0.79, 95%CI: −0.89, −0.69), (MD = −0.79, 95%CI: −0.88, −0.70), (MD = −0.97, 95%CI: −1.05, −0.89), (MD = −0.81, 95%CI: −0.86, −0.76). 1 study mentioned the measurement of SAS (MD = −4.18, 95%CI: −5.75, −2.61). 1 study mentioned the measurement of SDS (MD = −3.33, 95%CI: −4.94, −1.72). 1 study mentioned the measurement of PSQI (MD = −0.92, 95%CI: −1.12, −0.72). 1 study mentioned the measurement of YDFPS (MD = −3.13, 95%CI: −3.61, −2.65). 2 studies mentioned the measurement of KMI (MD = −3.83, 95%CI: −4.56, −3.11). 1 study mentioned the measurement of QL (MD = 14.00, 95%CI: 8.31, 19.69), These indicators all showed that the curative effect had been significantly enhanced after adding Hebaihe Dihuang Decoction(*P* < 0.00001). 1 study mentioned the measurement of HAMD (MD = −1.34, 95%CI: −2.49, −0.19). 1 study mentioned the measurement of HAMA (MD = −1.87, 95%CI: −3.32, −0.42), These two indicators also showed a better curative effect, as shown in Table 5.

#### 5.2.3. The Effects of Peripheral Serum Hormones, Vasomotor Factors and Immune Function of Conventional Treatment Drugs and Conventional Treatment Drugs Combined with Baihe Dihuang Decoction in the Treatment of Menopausal Syndrome

Analysis indicators are LH, FSH, E_2_, T, PRL, NE, 5-HT, NO, ET-1, CGRP, CD-3, CD-4, CD-8, IL-2.2 studies mentioned the determination of LH (MD = −6.94, 95% CI: −10.93, −2.96), 2 studies mentioned the determination of FSH (MD = −9.59, 95% CI: −18.44, −0.73), 2 studies mentioned the determination of E_2_ (MD = 56.53, 95% CI: −23.99, 137.05), The results showed that combined Baihe Dihuang Decoction could reduce the levels of LH and FSH, and the levels of E_2_, but the differences were not statistically significant (*P*=0.0006), (*P*=0.03) and (*P*=0.17). 1 study mentioned the determination of T (MD = 9.49, 95% CI: 9.40, 9.57), Indicates that the *T* level is increased and statistically significant (*P* < 0.00001). 1 study mentioned the measurement of PRL (MD = −0.22, 95%CI: −28.10, 27.66). 1 study mentioned the determination of NE (MD = 23.42, 95%CI: 17.79, 29.05), 1 study mentioned the determination of 5-HT (MD = 0.71, 95% CI: 0.58, 0.84), Showed that both NE and 5-HT levels had enhanced and were statistically different (*P* < 0.00001). 1 study mentioned the determination of NO (MD = 9.29, 95%CI: −140.61, 159.19). 1 study mentioned the determination of ET-1 (MD = −8.13, 95%CI: −10.51, −5.75) was statistically different (*P* < 0.00001), 1 study mentioned the determination of CGRP (MD = −5.20, 95%CI: −6.42, −3.98) was statistically different (*P* < 0.00001). 2 studies mentioned the determination of CD-3 (MD = 6.11, 95%CI: 5.35, 6.86), 2 studies mentioned the determination of CD-4 (MD = 7.36, 95%CI: 6.43, 8.28), 2 studies mentioned the determination of CD-8 (MD = −5.59, 95%CI:−6.80, −4.37), 2 studies mentioned the determination of IL-2 (MD = 10.39, 95%CI: 5.76, 15.01). It showed that after combining with Baihe Dihuang Decoction, CD-3, CD-4 and IL-2 were significantly enhanced, and CD-8 was significantly diminished (*P* < 0.00001), as shown in Table 6. Insomnia.

### 5.3. Quality of Included Trials

**Figure 5:**
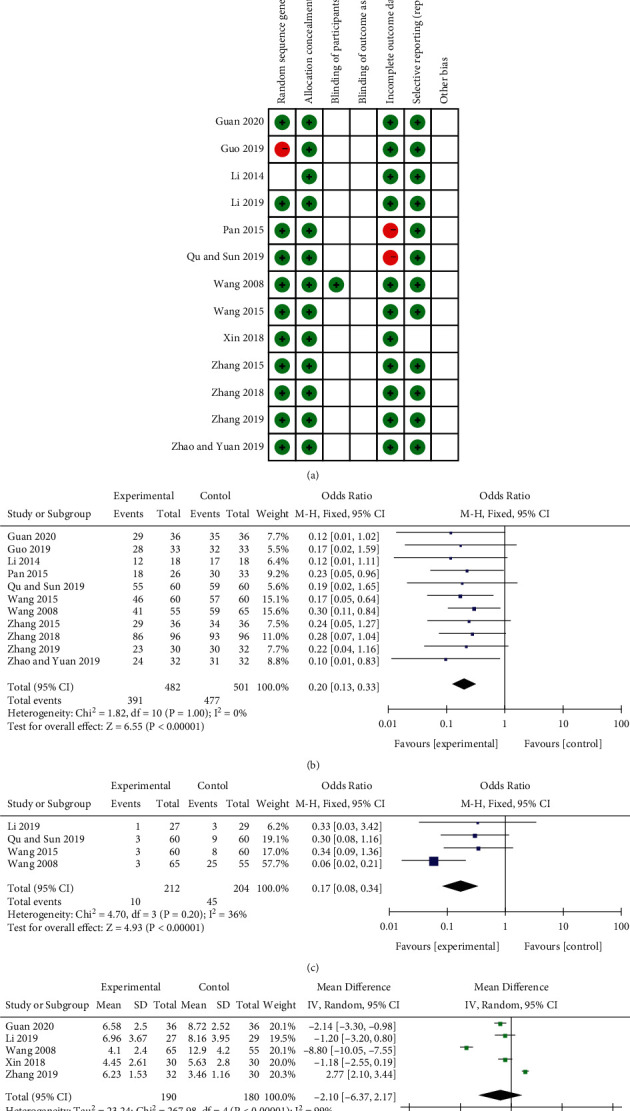
Insomnia correlation charts. Note: (a) Bias assessment risk of the study. Red circle, high bias risk; green circle, low bias risk; blank, unclear bias risk. (b) Forest chart of the total effective rate in the treatment of insomnia. (c) Forest chart of incidence of adverse reactions in the treatment of insomnia. (d) Forest plot of PSQI value for insomnia treatment.

According to Cochrane's risk of bias estimates, 11 trials mentioned randomly assigned participants and 1 did not mention it. 1 article did not randomly assign participants. 1 article mentioned blindness of the subject, and the other 12 did not mention it. All studies had a low risk of allocation concealment. All studies did not mention blinding result evaluation. 11 trials all had low data integrity risks. 12 trials had a low risk of selective reporting, as shown in Figure 5(a).

### 5.4. Analytical Result Measurement

#### 5.4.1. Total Effective Rate and Incidence of Adverse Reactions of Conventional Treatment Drugs and Conventional Treatment Drugs Combined with Baihe Dihuang Decoction in the Treatment of Insomnia

Judgement criteria for overall efficiency: Significantly effective: fall asleep fast, sleep quality is good, and nerve function is normal; Effective: fall asleep faster, sleep quality is average, and nerve function is basically normal; Invalid: slow falling asleep, poor sleep quality, severe neurological deficits. Healed and effective are included in the total effective. The overall effective rate refers to the proportion of patients receiving rehabilitation and efficacy evaluation in the total group. 11 studies reported the total effective rate. The fixed-effect model (*P*=1.00, *I*^2^ = 0%) meta-analysis results showed that the combination of Baihe Dihuang Decoction in the treatment of insomnia could significantly augment the efficacy (MD = 0.20, 95%CI: 0.13, 0.33; *P* < 0.00001), as shown in Figure 5(b). 4 studies provided descriptions of adverse reactions after treatment with commonly used anti-insomnia drugs, such as dreaminess, irritability, dry mouth and lack of energy. The fixed-effect model (*P*=0.20, *I*^2^ = 36%) meta-analysis results showed that the combination of Baihe Dihuang Decoction in the treatment of insomnia could significantly diminish the incidence of adverse reactions (MD = 0.17, 95%CI: 0.08, 0.34; *P* < 0.00001), as shown in Figure 5(c).

#### 5.4.2. PSQI of Conventional Treatment Drugs and Conventional Treatment Drugs Combined with Baihe Dihuang Decoction in the Treatment of Insomnia

PSQI is an important indicator reflecting the sleep status of patients with insomnia included in the study. Five literature reported the detection of PSQI indicators. Random effects model (*P* < 0.00001, *I*^2^ = 99%) meta-analysis results showed that combining Baihe Dihuang Decoction could reduce PSQI in the treatment of depression (MD = −2.10, 95%CI: −6.37, 2.17; *P*=0.33), as shown in Figure 5(d).

#### 5.4.3. Other Score Indexes of Conventional Treatment Drugs and Conventional Treatment Drugs Combined with Baihe Dihuang Decoction in the Treatment of Insomnia

TCM symptom score (TSS), sleeping time (ST), falling asleep time (FAT), number of night wakes (NNW), sleep efficiency (SE), sleep quality (SQ), dreaminess or nightmares (DN), Drowsiness, lack of energy (LE), HAMD, HAMA, AIS-8, ASRSA, GSP are various scoring indicators used to detect the degree of insomnia in patients. 2 studies mentioned the determination of TSS (MD = −1.70, 95%CI: −2.69, −0.70), there is a statistical difference (*P*=0.009). 2 studies mentioned the determination of ST (MD = 0.62, 95% CI: −2.28, 3.52). 2 studies mentioned the determination of FAT (MD = −9.03, 95%CI: −20.21, 2.15). 2 studies mentioned the determination of NNW (MD = −1.09, 95%CI: −1.34, −0.84), which is statistically significant (*P* < 0.00001). 1 study mentioned SE measurement (MD = 21.00, 95%CI: 11.12, 30.88), which is statistically significant (*P* < 0.00001). 1 study mentioned SQ determination (MD = −8.32, 95%CI: −8.48, −8.16) was statistically significant (*P* < 0.00001). 1 study mentioned that the determination of DN (MD = −0.60, 95%CI: −0.96, −0.24) was statistically significant (*P*=0.001). 1 study mentioned that the measurement of Drowsiness (MD = −1.70, 95%CI: −2.00, −1.40) is statistically significant (*P* < 0.00001). 1 study mentioned that the measurement of LE (MD = −1.20, 95%CI: −1.53, −0.87) was statistically significant (*P* < 0.00001). 1 study mentioned that the measurement of HAMD (MD = −1.25, 95%CI:−2.03, −0.47) was statistically significant (*P*=0.002). 1 study mentioned that the determination of AIS−8 (MD = −1.49, 95%CI:−2.30, −0.67) was statistically significant (*P*=0.0008). 1 study mentioned that ASRSA's determination (MD = −2.14, 95%CI:−3.30, −0.98) was statistically significant (*P*=0.0003). 1 study mentioned the measurement of GSP (MD = −2.58, 95%CI:−18.01, 12.85), as shown in Table 7. The results showed that insomnia was enhanced after combined Baihe Dihuang Decoction.

### 5.5. Publication Bias

**Figure 6:**
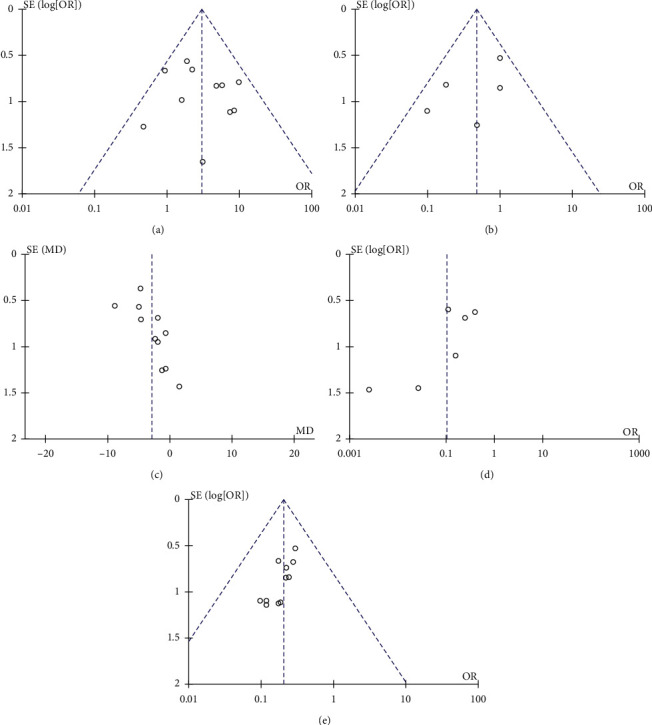
Funnel plot of each treatment index.

The publication bias is represented by a funnel chart. List in turn the total effective rate of conventional anti-depressant drugs combined with Baihe Dihuang Decoction for conventional treatment of depression, the incidence of adverse reactions, and the funnel chart of HAMD, as shown in Figures 6(a)–6(c). Funnel chart of the total effective rate of conventional anti-menopausal syndrome drugs combined with Baihe Dihuang Decoction and conventional anti-menopausal syndrome drugs, as shown in Figure 6(d). A funnel chart of the overall effectiveness of conventional anti-insomnia drugs combined with Baihe Dihuang Decoction and conventional anti-insomnia drugs, as shown in Figure 6(e). Most of the researches were concentrated in the middle and upper part of the funnel diagram, which were generally funnel-shaped and symmetrical. There was no significant publication bias.

## 6. Discussion

The World Health Organization (WHO) report shows that as of 2015, there are as many as 322 million depressed patients worldwide, the global prevalence rate of depression is about 4.4%, and the prevalence rate in my country is about 4.2%. Depression is the world's fourth most common disease and is expected to become the second most common disease by 2030 [34]. Depression is the disease with the heaviest burden among non-communicable diseases. It is a kind of psychotic mood disorder. Its main clinical feature is long-lasting and significant depression. The main performance for negative emotions, slow thinking, loss of will, cognitive impairment, pessimism and even more likely to commit suicide [35]. Therefore, how to improve the cognitive function of patients with depression, maintain the treatment effect, relieve and control the symptoms of depression, and avoid the recurrence of depression is the key topic of clinical research [36]. The common pathogenesis of depression may be because the insufficiency of neurotransmitters such as serotonin and norepinephrine in the brain is the main factor leading to depression, and dopamine is also involved in the pathogenesis of depression, 5HT and norepinephrine reuptake inhibitors are often used clinically to treat depression, and depression is treated by increasing the content of neurotransmitters such as 5-HT and norepinephrine [37]. Although these drugs have good anti-anxiety and anti-depressant effects, they also have side effects such as arrhythmia [38]. Patients are often accompanied by endocrine dysfunction, which can be manifested in a decrease in E_2_ levels and an increase in FSH and LH levels. Modern pharmacological studies have shown that Baihe Dihuang Decoction can increase the levels of dopamine and 5-HT in the body. After combining with Baihe Dihuang Decoction, the levels of 5-HT, NE, FSH and E_2_ in the patient's body can be increased, and the content of FSH decreased, indicating that it can be improved Endocrine disorders to treat depressed patients. Comparing conventional treatment combined with Baihe Dihuang Decoction and conventional treatment of HAMD, Kupperman, NIHSS and other evaluation indicators can show that the combination of Baihe Dihuang Decoction has a better therapeutic effect, and can significantly improve the total effective rate and reduce the incidence of adverse reactions. Therefore, it can be concluded that compared with the use of conventional drugs alone, the combination of Baihe Dihuang Decoction on the basis of it has a positive effect on the treatment and prognosis of patients with depression.

Foreign research shows that about 78% of women will have menopausal symptoms. Domestic research has found that the incidence of menopausal syndrome now fluctuates in the range of 50% to 80%. According to the survey, women have the highest incidence rate between 51 and 55 years old, and the degree of symptoms reaches moderate to severe.At the same time, if the patient is accompanied by other underlying diseases, the incidence is even higher, up to 64% [39]. During menopause, the gonads in the human body begin to shrink, and the original endocrine function begins to undergo a series of changes [40], In addition to the more typical symptoms, most menopausal women will have obvious neuropsychiatric symptoms, which are mainly manifested as suspiciousness, memory loss, emotional instability, and decreased work ability [16], Some women also often have a series of visceral functions. Disordered symptoms, collectively referred to as “menopausal syndrome” in medicine [41]. The common pathogenesis of menopausal syndrome may be caused by the imbalance of H–P–O axis neuroendocrine function after ovarian function decline, and the decrease of serum monoamine transmitters [42]. At present, drugs such as estradiol, norethindrone acetate, oryzanol are generally used clinically [43]. Although these drugs have achieved certain effects, the overall effect is average and cannot achieve the expected results of patients. Therefore, more effective and reasonable treatment methods need to be studied in depth in order to improve hormone levels, relieve clinical symptoms, and improve quality of life. The vasomotor factor NO and ET-1 are reproductive hormone regulatory peptides, which have a good regulatory effect on the thalamus-pituitary-ovarian axis (HPOA) [42], and the disorder of HPOA can be manifested by abnormal levels of NE and 5-HT. After combining with Baihe Dihuang Decoction, the levels of NO, NE, 5-HT in the patient's body increased, and the content of ET-1 decreased, indicating that it can regulate HPOA and improve the symptoms of menopausal syndrome. Improve immune function by increasing the content of CD-3, CD-4 and IL-2. Comparing conventional treatment combined with Baihe Dihuang Decoction and conventional treatment of MENQOL, Kupperman and other evaluation indicators can show that the combination of Baihe Dihuang Decoction has a better therapeutic effect and can improve the overall effective rate. Therefore, it can be concluded that the combination of Baihe Dihuang Decoction has a positive effect on the treatment of menopausal syndrome patients compared with the conventional drugs alone.

The global incidence of insomnia is 10% to 50%, and it is currently the sleep disorder with the highest prevalence rate. The World Health Organization estimates that there are nearly 700 million people suffering from insomnia worldwide. Insomnia is a sleep initiation disorder or sleep maintenance disorder that occurs repeatedly when there is sufficient sleep time and a good sleep environment [44]. It is often characterized by the inability to get normal sleep, and it usually becomes difficult to fall asleep, dreamy, easy to wake up, etc [45]. Long-term insomnia can lead to a series of health and psychological problems, which can induce heart and brain diseases, diabetes, etc [46]. It can be accompanied by depression, which seriously affects the patient's health and quality of life [47]. The common pathogenesis of insomnia may be caused by the enhanced activity of the hypothalamic-pituitary-adrenal (HPA) axis and the changes of cytokines such as interleukin (IL) and tumor necrosis factor (TNF), At present, the treatment of insomnia with anxiety in modern medicine mainly focuses on improving sleep quality and anti-anxiety treatment, and mostly uses sedative hypnosis and anti-anxiety drugs [48]. But its therapeutic effect is not good. In recent years, Chinese medicine has made some progress in the treatment of insomnia, and obtained good treatment results. Baihe Dihuang Decoction works by modulating pathways related to the nervous system, endocrine system, inflammation and immune system Comparing the PSQI, ZYZZJF, SMSJ and other evaluation indicators of conventional treatment combined with Baihe Dihuang Decoction and conventional treatment can show that the combination of Baihe Dihuang Decoction has a better therapeutic effect, and can significantly improve the total effective rate while significantly reducing the incidence of adverse reactions. Therefore, it can be concluded that the combination of Baihe Dihuang Decoction has a positive effect on the treatment and prognosis of patients with insomnia compared with the use of conventional drugs alone.

From the above discussion, it is easy to find that depression, menopausal syndrome and insomnia are all neuropsychiatric disorders, and their common pathological processes involve various aspects of neurotransmitter release, anti-inflammatory and immune regulation. The mechanism of action of Baihe Dihuang Decoction includes promoting monoamine neurotransmitter release, inhibiting monoamine oxidase activity, regulating hypothalamic-pituitary-adrenal (HPA) axis function, anti-inflammation, immunomodulation and neuroprotection, etc. These findings provide some scientific basis for future pharmacological studies in the treatment of depression, menopausal syndrome and insomnia with Baihe Dihuang Decoction. The results of Meta-analysis also showed that the combination of Baihe Dihuang Decoction for the treatment of depression, menopausal syndrome and insomnia could improve the efficacy and reduce the incidence of adverse reactions, which is worthy of clinical promotion and application.

The limitations of Meta are as follows: (1) Among the included 44 articles, only one mentions allocation concealment, and none of the studies mentions blindness and outcome evaluation of subjects, which is likely to lead to a certain degree of bias. Since this study only included Chinese literature and did not include literature in other languages, it will have a certain impact on the comprehensiveness of the research. (2) There is no uniform standard for the dosage of Baihe Dihuang Decoction, the treatment period and the evaluation standard of patients' curative effect. (3) All the included literature lacks the observation of long-term curative effect, and the index such as recurrence rate is not used as one of the curative effect evaluation criteria, and its long-term effect and curative effect cannot be clarified. (4) Most of the included studies are small sample trials and there are differences in race, ethnicity, and regional conditions, and lack of representativeness. It is necessary to increase the sample size to make the research results closer to the overall authenticity.

## Figures and Tables

**Table 1:** Basic characteristics and quality evaluation of literaturs.

Disease	Author, year	Cases *T*/*C*	Diagnostic standard	Age (years) range, mean		Sex male/female	
Depressed	Meng and Zhang, 2020	35/35	CCMD-2&CDEES&HAMD	*T*; 29∼77, 53.18 ± 8.16	*C*; 29∼78, 53.75 ± 8.25	*T*; 21/14	*C*; 20/15
	Duan et al., 2020	38/38	HAMD	*T*; 49∼59, 53.53 ± 2.12	*C*; 48∼58, 53.21 ± 2.18	NR	NR
	Zhang et al., 2019	44/44	HAMD&NIHSS&QOL-100	*T*; 54∼79, 64.12 ± 2.46	*C*; 53∼79, 64.12 ± 2.46	*T*; 29/15	*C*; 28/16
	Guo et al., 2016	43/43	KMI&HAMD&psychiatry&GCM	*T*; 40∼55, 39.5 ± 6.0	*C*; 42∼55, 38.4 ± 7.2	NR	NR
	Wang et al., 2014	60/40	HADS	NR	NR	NR	NR
	Yin et al., 2014	18/18	HAMD	NR	NR	NR	NR
	Li and Gao, 2014	34/34	CMD&CCMD-3	*T*; 35∼70, 51.2 ± 3.5	*C*; 35∼70, 51.2 ± 3.5	*T*; 20/14	*C*; 18/16
	Tang and Bu, 2012	26/26	NIHSS	NR	NR	NR	NR
	Miu et al., 2012	19/19	HAMD	*T*; 72.6 ± 13.2	*C*; 73.3 ± 12.7	*T*; 6/13	*C*; 7/12
	Nie et al., 2010	33/29	HAMD	*T*; 65∼84	*C*; 63∼85	*T*; 14/19	*C*; 16/14
	Chen et al., 2004	40/39	HAMD&AFI	NR	NR	NR	NR
	Wang et al., 2015	30/30	HAMD&KMI	NR	NR	NR	NR
	Wen et al., 2018	31/32	HAMD	*T*; 61∼83, 71.7 ± 4.7	*C*; 60∼83, 71.2 ± 4.6	*T*; 18/14	*C*; 17/17

Menopausal syndrome	Xie et al., 2020	30/30	AFG&IMCM	*Y*; 63.87 ± 8.05	*C*; 63.77 ± 2.404	—	
	Han and Wan, 2020	67/66	OG&GDTCGDTCM	*T*; 45∼55, 50.27 ± 6.35	*C*; 45∼55, 50.64 ± 6.58		
	Shen et al., 2019	50/50	NR	*T*; 43∼55, 44 ± 8	*C*; 40∼55, 45 ± 10		
	Song et al., 2018	30/30	GPTTDC&HAMD&CDTE&GPCRNCM	*T*; 40∼60, 50 ± 3.8	*C*; 45∼55, 49 ± 4.1		
	Zhou et al., 2015	86/86	NR	T; 42∼53, 45.27 ± 10.12	*C*; 43–55, 43.78 ± 11.65		
	Zhang et al., 2012	27/27	DSM&PCMP	*T*; 43∼54, 46.5 ± 6.3	*C*; 44∼55, 45.3 ± 7.2		

Insomnia	Guan et al., 2020	36/36	HAMD&PSQI&ASRSA	*T*; 42∼65, 49.7 ± 6.4	*C*; 41∼65, 49.4 ± 6.8	*T*; 17/19	*C*; 16/20
	Li et al., 2019	27/29	CCMD-3&GPCRNCM	*T*; 51.85 ± 7.49	*C*; 52.03 ± 7.10	*T*; 12/15	*C*; 12/17
	Zhang et al., 2019	32/30	CCCMD&GPCRNCM&RPTITSD	*T*; 30∼55, 41.2 ± 3.6	*C*; 30∼55, 40.8 ± 4.1	*T*; 15/17	*C*; 14/16
	Guo et al., 2019	33/33	CDTE	T; 62∼79 66.52 ± 1.11	*C*; 60∼78 66.23 ± 1.25	*T*; 14/19	*C*; 15/18
	Zhao and Yuan, 2019	32/32	NR	T; 51∼87 68.42 ± 0.25	*C*; 50∼88 68.39 ± 0.57	*T*; 17/15	*C*; 18/14
	Xin et al., 2018	30/30	CDCMDC&CJCTCM	*T*; 42.2 ± 6.54	*C*; 40.7 ± 4.95	T; 12/18	*C*; 11/19
	Zhang et al., 2018	96/96	Endocrinology&CCMD	*T*; 46∼56	*C*; 43∼58	NR	NR
	Wang et al., 2015	60/60	CCMD-3	NR	NR	NR	NR
	Qu and Sun, 2019	51/41	urosurgery&IMCM	*T*; 32∼80, 54.1 ± 3.3	*C*; 29∼74, 57.4 ± 2.7	*T*; 36/24	*C*; 35/25
	Li et al., 2014	18/18	CDTE	*T*; 66.5 ± 5.6	*C*; 67.1 ± 5.3	*T*; 7/11	*C*; 6/12
	Wang et al., 2008	65/55	CCMD&HAMD&CDTEIM	NR	NR	*T*; 34/31	*C*; 23/32
	*Pan* et al., 2015	38/36	CCCMD	*T*; 36∼47, 42.73 ± 4.27	*C*; 38∼48, 43.64 ± 3.76	NR	NR
	Zhang et al., 2015	36/36	OG&CCMD	*T*; 49.7 ± 6.4	*T*; 69.4 ± 6.8	NR	NR

*Note*: CCMD, chinese classification of mental disorders; CCMD-2, chinese classification of mental disorders vertion 2; CCMD-3, chinese classification of mental disorders vertion 3; CDEES, criteria for diagnosis and efficacy evaluation of stroke; HAMD, hamilton depression scale; NIHSS, national institutes of health stroke scale; QOL-100, quality of life-100; KMI, kupperman; GCM, gynecology of Chinese medicine; HADS, hospital anxiety and depression scale; CMD, chinese medicine diagnostics; AFI, assessment of functional impairment; AFG, atrial fibrillation guide vertion 2014; IMCM, internal medicine of chinese medicine; OG, obstetrics and gynecology; GDTCGDTCM, guidelines for the diagnosis and treatment of common gynecological diseases in traditional Chinese medicine; GPTTDC, guidelines for prevention and treatment of type 2 diabetes in china vertion 2013; CDTE, criteria for diagnosis and therapeutic effect; GPCRNCM, guiding principles for clinical research of new chinese medicines; DSM, the diagnostic and statistical manual of mental disorders; PCMP, practical chinese medicine psychiatry; PSQI, pittsburgh sleep quality index; ASRSA, asberg side-effect rating scale for antidepressant; CCCMD, Chinese classification of mental disorders; RPTITSD, research progress in the treatment of insomnia by TCM syndrome differentiation; CDCMDC, classification and diagnostic criteria of mental disorders in China; CJCTCM, classification and judgment of constitution of traditional Chinese medicine; CDTEIM, criteria for diagnosis and therapeutic effect of internal medicine.

**Table 2:** Treatment protocols and indicators.

Disease	Study ID (Author, year)	Treatment group	Contral group	Duration/follow measure	Outcome measure
Depressed	Duan et al., 2020	FMT + BDD	FMT	8 week/NR	HAMD, KMI, NE, 5-HT, TER, IAR
	Meng and Zhang, 2020	Fluoxetine + BDD	Fluoxetine	8 week/NR	HAMD, SDSS, NIHSS, TER, IAR
	Zhang et al., 2019	Paroxetine + BDD	Paroxetine	8 week/NR	HAMD, NIHSS, QOL-100, TER, IAR
	Guo et al., 2016	FMT + BDD	FMT	6 week/NR	HAMD, PSQI, 5-HT, NE, TER, FSH, LH, LH, E_2_
	Li and Gao, 2014	Fluoxetine + BDD	Fluoxetine	6 week/NR	HAMD, TER, STCM(AS, SD, Despair)
	Wang et al., 2014	Vitamins + Ginkgo + BDD	Vitamins + Ginkgo	4 week/NR	HADS, TER
	Miu et al., 2012	GJS + BDD	FMT	6 week/NR	HAMD, TER
	Tang and Bu, 2012	Fluoxetine + BDD + GJS	Fluoxetine	2 week/6 m	NIHSS, OHS, ADL, PSD
	Nie et al., 2010	BDD	Paroxetine	8 week/NR	HAMD, TER, IAR
	Chen et al., 2004	BDD	Paroxetine	4 week/NR	ADL, HAMD, NDS
	Yin et al., 2014	GJS + BDD	FMT	6 week/NR	HAMD, TER
	Wang et al., 2015	GJS + BDD	Duloxetine	8 week/NR	HAMD, KMI, TER, IAR
	Wen et al., 2018	GJS + BDD	Fluoxetine	6 week/NR	HAMD, TER

Menopausal syndrome	Xie et al., 2020	GJS + BDD	Oryzanol	4 w/NR	KMI, TER
	Shen et al., 2019	EV + BDD	EV	3 m/NR	CD-(3, 4, 8), IL-2, TER, QL
	Song et al., 2018	Metformin + FMT + BDD	Metformin + FMT	60 d/NR	HAMD, HAMA, TER, C-, 348
	Zhou et al., 2015	Nilestriol + BDD	Nilestriol	4 w/NR	CD-(3, 4, 8), IL-2, TER, LH, FSH, *E*_2_, T, PRL
	Zhang et al., 2012	Paroxetine + BDD	Paroxetine	4 w/NR	TER
	Han and Wan, 2020	Estradiol + BDD + HES	Estradiol	3 m/NR	KMI, MENQOL(BC, psychological, body, sex), SAS, SDS, PSQI, E2, FSH, LH, NE, 5-HT, NO, ET-1, CGRP, TER, YDFPS

Insomnia	Guo et al., 2019	SS + BDD	SS	2 w/NR	TER
	Zhao and Yuan, 2019	Paroxetine + BDD	Paroxetine	3 w/NR	NIHSS, TER, SQ
	Xin et al., 2018	BDD	Zopiclone	2 w/NR	PSQI
	Zhang et al., 2018	BDD + BEP	Estazolam	4 w/NR	TER, FAT, ST, SD, NNW
	Zhang et al., 2015	BDD + YJW	Estazolam	4 w/NR	TER
	*Pan* et al., 2015	Bupleurum + KOS + BDD	ZZ	2 w/1 m	AIS-8, TER
	Wang et al., 2015	Acupuncture + BDD	Diazepam + Diethylstilbestrol	3 w/NR	TER, IAR
	Li et al., 2014	SS + BDD	NR	2 w/NR	TER
	Guan et al., 2020	Trazodone + BDD	Trazodone	3 m/1m	HAMD, PSQI, ASRSA, TER
	Li et al., 2019	BDD + EA	Zopiclone	28 d/NR	PSQI, HAMA, GSP, IAR, TSS
	Zhang et al., 2019	BDD	Alprazolam	30 d/NR	PSQI, TER, TSS
	Qu and Sun, 2019	BDD + SS	Estazolam	2 w/NR	TER, IAR
	Wang et al., 2008	BDD	Alprazolam	12 w/NR	PSQI, TER, AR, FAT, ST, SE, DN, Drowsiness

*Note.* HAMD, hamilton depression scale; KMI, kupperman; NE, norepinephrine; TER, total effective rate; IAR, incidence of adverse reactions; SDSS, social dysfunction scale; NIHSS, national institutes of health stroke scale; QOL-100, quality of life-100; PSQI, pittsburgh sleep quality index; FSH, follicle stimulating hormone; LH, luteinizing hormone; E2, estradiol; STCM, symptoms of traditional Chinese medicine; AS, anxiety somatization; SD, sleep disorder; HADS, hospital anxiety and depression scaleor; OHS, social function evaluation; ADL, activity of daily living; PSD, post-stroke depression; NDS, neurological deficit score; T lymphocyte CD subgroup, CD-(3, 4, 8); QL, quality of life; HAMA, hamilton anxiety scale; T, testosterone; PRL, prolactin; MENQOL, menopause-specific quality of life questionnaire; BC, blood; circulation; SAS, simpson-angus scale; SDS, anxiety self-rating scale; NO, nitric oxide; ET-1, endothelin-1; CGRP, calcitonin gene-related peptide; YDFPS, yin deficiency and fire prosperity syndrome; SQ, sleep quality; FAT, falling asleep time; ST, sleeping time; SD, sleep depth; NNW, number of night wakes; AIS-8, athens insomnia scale; ASRSA, asberg side-effect rating scale for antidepressant; GSP, glycosylated serum protein; TSS, TCM symptom score; SE, sleep efficiency; DN, dreaminess or nightmares, LE, lack of energy; FMT, flupenthixol and meritroxine; BDD, baihe dihuang decoction; GJS, ganmai jujube soup; EV, estradiol valerate; HES, huanglian ejiao soup; SS, suanzaoren soup; zopiclone; BEP, beans in ear points; YJW, yueju wan; KOS, keel oyster soup; EA, ear acupuncture; ZZ, zolpidem tartrate.

**Table 3:** Other scoring indicators used to evaluate depression.

Index	Numer of studies	Study ID (Author, year)	Cases of experimental group	Cases of control group	MD [95%CI]	Z-value	*P* value
KMI	2	Duan et al., 2020 Wang et al., 2015	68	68	−5.41 [−10.28, −0.54]	2.18	0.03
NIHSS	2	Yang and Zhang, 2020 Zhang et al., 2019	79	79	−7.18 [−8.88, −5.48]	8.26	<0.00001
ADL	2	Tang and Bu, 2012 Chen et al., 2004	66	65	3.83 [−7.84, 15.50]	0.64	0.52
QOL-100	1	Zhang et al., 2019	44	44	14.79 [13.22, 16.36]	18.41	<0.00001
AS	1	Li and Gao, 2014	34	34	−1.92 [−2.45, −1.39]	7.13	<0.00001
SD	1	Li and Gao, 2014	34	34	−2.17 [−2.45, −1.88]	15.08	<0.00001
Despair	1	Li and Gao, 2014	34	34	−0.08 [−0.32, 0.17]	0.61	0.54
OHS	1	Tang and Bu, 2012	26	26	−0.81 [−1.41, −0.21]	2.65	0.008
PSD	1	Tang and Bu, 2012	26	26	0.32 [0.09, 1.13]	1.77	0.08
NDS	1	Chen et al., 2004	40	39	0.20 [−1.93, 2.33]	0.18	0.85
PSQI	1	Guoet et al., 2016	43	43	−2.70 [−3.15, −2.25]	11.78	<0.00001
SDSS	1	Yang and Zhang, 2020	35	35	−3.50 [−4.31, −2.69]	8.48	<0.00001

**Table 4:** Analysis of neuroendocrine function index table in the treatment of depression.

Index	Numer of studies	Study ID (Author, year)	Cases of experimental group	Cases of control group	MD [95%CI]	*Z*-value	*P* value
5-HT	2	Guo et al., 2016 Duan et al., 2020	81	81	33.80 [15.60, 51.99]	3.64	0.003
NE	2	Duan et al., 2020 Guo et al., 2016	81	81	20.70 [12.66, 28.74]	5.04	<0.00001
FSH	1	Guo et al., 2016	43	43	−1.10 [−16.91, 14.71]	0.14	0.89
LH	1	Guo et al., 2016	43	43	0.30 [−7.36, 7.96]	0.08	0.94
E_2_	1	Guo et al., 2016	43	43	3.90 [−8.52, 16.32]	0.62	0.54

**Table 5:** Other scoring indicators used to evaluate menopausal syndrome.

Index	Numer of studies	Study ID (Author, year)	Cases of experimental group	Cases of control group	MD [95%CI]	*Z*-value	*P* value
MENQOL (BC)	1	Han and Wan 2020	66	67	−0.79 [−0.89, −0.69]	14.79	<0.00001
MENQOL (psychological)	1	Han and Wan 2020	66	67	−0.79 [−0.88, −0.70]	16.71	<0.00001
MENQOL (body)	1	Han and Wan 2020	66	67	−0.97 [−1.05, −0.89]	23.69	<0.00001
MENQOL (sex)	1	Han and Wan 2020	66	67	−0.81 [−0.86, −0.76]	31.7	<0.00001
SAS	1	Han and Wan 2020	66	67	−4.18 [−5.75, -2.61]	5.21	<0.00001
SDS	1	Han and Wan 2020	66	67	−3.33 [−4.94, −1.72]	4.04	<0.00001
PSQI	1	Han and Wan 2020	66	67	−0.92 [−1.12, −0.72]	9.02	<0.00001
YDFPS	1	Han and Wan 2020	66	67	−3.13 [−3.61, −2.65]	12.88	<0.00001
KMI	2	Han and Wan 2020 Xie 2020	96	97	−3.83 [−4.56, −3.11]	10.35	<0.00001
QL	1	Shen 2019	50	50	14.00 [8.31, 19.69]	4.82	<0.00001
HAMD	1	Song 2018	30	30	−1.34 [−2.49, -0.19]	2.28	0.02
HAMA	1	Song 2018	30	30	−1.87 [−3.32, −0.42]	2.54	0.01

**Table 6:** Analysis of peripheral serum hormone, vasomotor factor and immune function in patients with menopausal syndrome.

Index	Numer of studies	Study ID (Author, year)	Cases of experimental group	Cases of control group	MD [95%CI]	*Z* value	*P* value
LH	2	Zhou 2015 Han and Wan 2020	152	153	−6.94 [−10.93, −2.96]	3.41	0.0006
FSH	2	Zhou 2015 Han and Wan 2020	152	153	−9.59 [−18.44, −0.73]	2.12	0.03
E_2_	2	Zhou 2015 Han and Wan 2020	152	153	56.53 [−23.99, 137.05]	1.38	0.17
T	1	Zhou 2015	86	86	9.49 [9.40, 9.57]	216.7	<0.00001
PRL	1	Zhou 2015	86	86	−0.22 [−28.10, 27.66]	0.02	0.99
NE	1	Han and Wan 2020	66	67	23.42 [17.79, 29.05]	8.16	<0.00001
5-HT	1	Han and Wan 2020	66	67	0.71 [0.58, 0.84]	11.12	<0.00001
NO	1	Han and Wan 2020	66	67	9.29 [−140.61, 159.19]	0.12	0.9
ET-1	1	Han and Wan 2020	66	67	−8.13 [−10.51, −5.75]	6.69	<0.00001
CGRP	1	Han and Wan 2020	66	67	−5.20 [−6.42, −3.98]	8.39	<0.00001
CD-3	2	Shen 2019 Zhou 2015	136	136	6.11 [5.35, 6.86]	15.92	<0.00001
CD-4	2	Shen 2019 Zhou 2015	136	136	7.36 [6.43, 8.28]	15.59	<0.00001
CD-8	2	Shen 2019 Zhou 2015	136	136	−5.59 [−6.80, −4.37]	9.02	<0.00001
IL-2	2	Shen 2019 Zhou 2015	136	136	10.39 [5.76, 15.01]	4.4	<0.0001

**Table 7:** Other scoring indicators used to evaluate insomnia.

Index	Numer of studies	Study ID (Author, year)	Cases of experimental group	Cases of control group	MD [95%CI]	*Z*-value	*PP* value
TSS	2	Li et al.,2019 Zhang et al., 2019	59	59	−1.70 [−2.69, −0.70]	3.34	0.0009
ST	2	Zhang et al., 2018 Wang et al., 2008	161	151	0.62 [−2.28, 3.52]	0.42	0.67
FAT	2	Zhang et al., 2018 Wang et al., 2008	161	151	−9.03 [−20.21, 2.15]	1.58	0.11
NNW	2	Zhang et al., 2018 Wang et al., 2008	161	151	−1.09 [−1.34, -0.84]	8.54	<0.00001
SE	1	Wang et al., 2008	65	55	21.00 [11.12, 30.88]	4.17	<0.0001
SQ	1	Zhao and Yuan., 2019	32	32	−8.32 [−8.48, −8.16]	100.41	<0.00001
DN	1	Wang et al., 2008	65	55	−0.60 [−0.96, −0.24]	3.27	0.001
Drowsiness	1	Wang et al., 2008	65	55	−1.70 [−2.00, −1.40]	11.04	<0.00001
LE	1	Wang et al., 2008	65	55	−1.20 [−1.53, −0.87]	7.17	<0.00001
HAMD	1	Guan et al., 2020	36	36	−1.25 [−2.03, −0.47]	3.15	0.002
AIS-8	1	*Pan* et al., 2015	38	36	−1.49 [−2.30, −0.67]	3.59	0.0003
ASRSA	1	Guan et al., 2020	36	36	−2.14 [−3.30, −0.98]	3.62	0.0003
GSP	1	Li et al., 2019	27	29	−2.58 [−18.01, 12.85]	0.33	0.74
